# Depression diagnosed by the mini international neuropsychiatric interview plus (MINI) in patients with chronic obstructive pulmonary disease: relationship with functional capacity and quality of life

**DOI:** 10.1186/s13104-016-1883-z

**Published:** 2016-02-04

**Authors:** Luciana de Carvalho Lopes Orlandi, José Felippe Pinho, Melissa Guarieiro Ramos Murad, Fábio Lopes Rocha, Maria Glória Rodrigues-Machado

**Affiliations:** Faculdade Ciências Médicas-Minas Gerais, Departamento de Fisioterapia, Belo Horizonte, Minas Gerais Brazil; Faculdade Ciências Médicas-Minas Gerais, Pós-Graduação, Alameda Ezequiel Dias, 275, 30130-110 Belo Horizonte, Minas Gerais Brazil; Faculdade Ciências Médicas-Minas Gerais. Departamento de Medicina, Belo Horizonte, Minas Gerais Brazil; Instituto de Previdência dos Servidores do Estado de Minas Gerais, Clínica de Psiquiatria, Belo Horizonte, Minas Gerais Brazil

**Keywords:** Depression, MINI, 6MWT, UULEX, SGRQ, COPD, Pulmonary function, Functional capacity

## Abstract

**Background:**

The prevalence of depression in patients with chronic obstructive pulmonary disease (COPD) is associated with a worsening of prognosis. Most studies classify COPD patients as depressive or non-depressive based on symptoms, rather than on a diagnosis using specific tools. Thus, the aim of this study was to determine the impact of depression, as diagnosed by the Mini International Neuropsychiatric Interview Plus (MINI), on functional capacity estimated by the 6-minute walk test (6MWT) and unsupported upper-limb exercise test, and quality of life estimated by Saint George’s Respiratory Questionnaire (SGRQ), among patients with COPD.

**Results:**

Using the MINI as a diagnostic tool, 22.2 % of all patients (6.6 % of all men and 41.6 % of all women) were diagnosed with depression. No significant differences were found between depressive and non-depressive patients with regard to anthropometric measurements, lung function, functional capacity, or quality of life variables. The best models for the dependent variables representing functional capacity and quality of life revealed that the covariates SGRQ_TOTAL_ and gender (R^2^ = 16.7 %) were significant in explaining the response variable for functional capacity of the upper limbs. Results also showed that age, monthly income, insomnia, and the results of a 6MWT were significant in explaining overall quality of life (R^2^ = 46 %), and that the percentage of the predicted forced expiratory volume in the first second post-bronchodilator and gender were significant in explaining walking distance (R^2^ = 22 %). Depression, as diagnosed by the MINI, was not significant in explaining any of the dependent variables.

**Conclusions:**

Despite a high prevalence of depression in COPD patients, especially in women, depression, as diagnosed by the MINI, was not correlated with functional capacity tests or quality of life in patients with moderate to very severe COPD in the present study. This suggests that depression identified by this diagnostic test may be more accurate than depression diagnosed by tests that evaluate symptoms, as they may be influenced by the perceptions of the patient in relation to their health.

## Background

By 2020, chronic obstructive pulmonary disease (COPD) is projected to cause over 6 million deaths annually, worldwide, thus becoming the third leading cause of death in the world [1]. Patients with COPD manifest systemic complications such as intolerance to exercise and diminished health-related quality of life, which are considered to be important causes of morbidity and mortality [1].

In addition to the systemic manifestations of COPD, the presence of comorbidities, such as depression, may also have negative impacts on physical performance and quality of life. Therefore, depression in COPD patients should be identified and accounted for in order to achieve successful intervention [2, 3].

According to the literature, more than one-third of COPD patients have symptoms of depression and anxiety, and the risk of depression is approximately 1.8-fold greater among patients with COPD than among individuals with no chronic comorbidities [4–6]. Factors that may contribute to this increase in the prevalence of depression include social isolation, frequent hospitalization, disease severity, physical disability in activities of daily living, poorer quality of life, and long-term oxygen therapy [3, 7].

Another factor that may be important in accounting for cases of depression among patients with COPD is the method used to identify depression. Despite numerous reports of a higher prevalence of depression in patients with COPD, it is important to recognize that most studies analyze the symptoms of depression without emphasizing the diagnosis [5, 8]. Therefore, this study aimed to analyze the influence of depression using a diagnostic tool, the mini international neuropsychiatric interview plus (MINI), rather than an analysis of the symptoms, to determine the effect of depression on functional capacity and quality of life among patients with COPD.

## Methods

A cross-sectional study was conducted with COPD patients from the pulmonology sector of the Hospital Governador Israel Pinheiro—Instituto de Previdência dos Servidores do Estado de Minas Gerais (HGIP/IPSEMG). Patients were evaluated over a period of 10 days by a physiotherapist, pulmonologist, and psychiatrist. The order in which patients underwent evaluations was random, and each evaluator was blind to the data generated by others.

### Ethical statement

This study was approved by the Human Research Ethics Committee of the Instituto de Previdência dos Servidores do Estado de Minas Gerais—Hospital Governador Israel Pinheiro—HGIP/IPSEMG (CAAE 0018.0.191.191-08 CEP 065/08), and all patients signed the terms, giving their informed consent. The information about participants’ identity was not included.

### Eligibility criteria

**Fig. 1:**
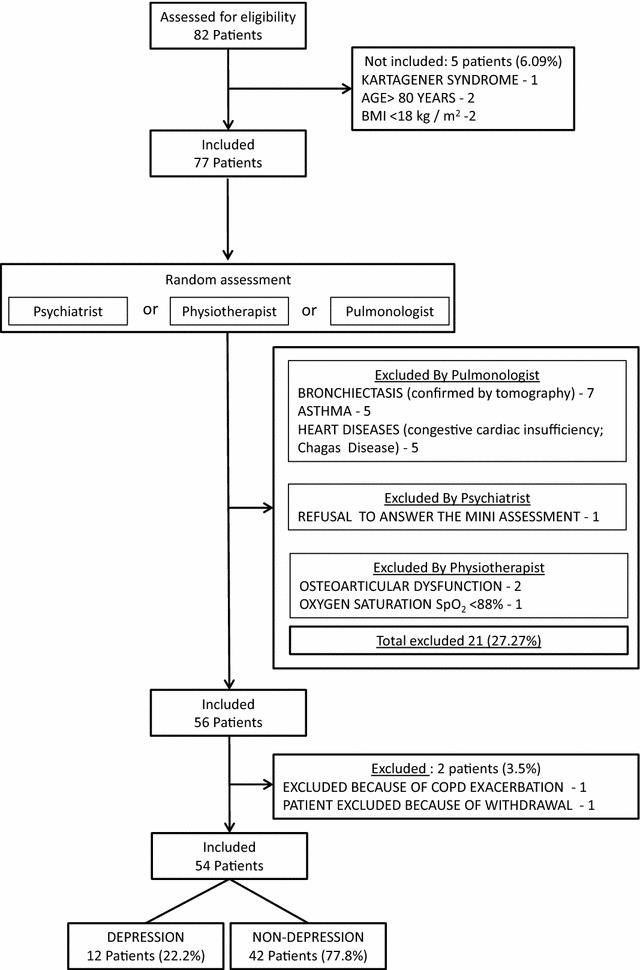
Flow diagram of COPD patients classified as depressive and non-depressive in this study

Patients were included if they had a diagnosis of COPD based on the guidelines established by the Global Initiative for chronic obstructive lung disease (GOLD), were over 40 years of age, and had been free of COPD exacerbations, according to the criteria of GOLD [1], for at least 8 weeks. Patients under prolonged oxygen therapy; those with severe comorbidities (an incapacitating or severe lung disease other than COPD, asthma, congestive heart failure, advanced chronic kidney failure, severe liver disease, cancer, insulin-dependent diabetes, psychosis, or dementia); those with a body mass index (BMI) below 18.5 kg/m^2^; and those with osseous, articular, and/or neuromuscular diseases that would limit performance on the functional capacity tests, were excluded from the study (Fig. 1).

### Assessment of socio-demographic and clinical data

The following socio-demographic and clinical data were recorded for all patients: age, height, weight, gender, schooling, marital status, income, smoking habits, use of alcohol, complaints of insomnia and daytime sleepiness, associated diseases, medication being taken, past history of depression, family history of depression, duration of COPD (years since diagnosis), number of hospitalizations in the previous year, and type of family support.

Lung function was assessed to diagnose COPD and determine its severity based on the standardized method recommended by the American Thoracic Society (ATS) [9] using a Vitatrace VT-130 SL^®^ spirometer (Pro Médico Ind Ltda). The parameters evaluated were forced vital capacity (FVC  % predicted), forced expiratory volume in the first second (FEV1 % predicted), and FVC/FEV_1_ (%). The reference equations used followed the guidelines for lung function tests proposed by Pereira and Neder [10].

### Psychiatric evaluation

Psychiatric evaluations consisted of a structured psychiatric interview using the MINI for the diagnosis of depression (depression_(MINI)_) [11], in addition to a brief, standardized, diagnostic interview, 15–30 min in duration. This evaluation was compatible with the diagnostic criteria established by the diagnostic and statistical manual of mental disorders (DSM-IV) and the international classification of diseases (ICD-10) [12, 13]. The MINI is designed for application in primary and psychiatric care, in both clinical practice and research, and can be used by clinicians after a training session of 1–3 h. It is organized into independent sections as a means of optimizing the sensitivity of the diagnostic tool. The MINI consists of 19 modules that explore 17 disorders of Axis I of the DSM-IV, as well as the risks of suicide and anti-social personality disorder. The MINI explores all inclusion and exclusion criteria and the chronology of 23 diagnostic categories of the DSM-IV, and has been proven to have satisfactory reliability. The MINI has psychometric properties similar to those of more complex standardized psychiatric interviews, such as the Composite International Diagnostic Interview and Structured Clinical Interview for DSM-III-R, Patient Version [11].

### Physical therapy assessment

#### Evaluation of functional capacity with the unsupported upper-limb exercise test (UULEX) and the six-minute walk test (6MWT)

The UULEX assesses the strength of the upper limb musculature by recording the number of elevations of the arm, whilst holding a plastic stick, in addition to the time spent performing this activity. A plastic stick with a weight of 0.2 kg, diameter of 25 mm, and a length of 0.84 m, was used initially. Upon reaching the maximum height from shoulder flexion, the rod was exchanged for another weighing 0.5 kg more, with subsequent increases of 0.5 kg every minute, to a maximum weight of 2 kg. This test is limited by symptoms of fatigue or dyspnea [14]. All patients performed the UULEX at least twice, with a 30-minute rest interval between each test. A third test was performed when the results of the first two tests differed by more than 10 %. The UULEX was ended when the patient presented with peripheral oxygen desaturation or tachycardia. The test with the greatest number of arm elevations was selected for analysis.

The 6MWT was performed based on the criteria of the ATS guidelines [15]. The test was implemented twice, with a 30-minute rest interval between tests [16]. A third test was performed when the results of the first two tests differed by more than 10 %. The test with the longest distance walked during 6 min (6MWD) was selected for analysis. Calculation of the predicted values were then performed using the reference equations for the 6MWD, as proposed by Enright and Sherrill [17].

Both before and after the UULEX and 6MWT, vital signs (blood pressure, respiratory rate (RR) and heart rate (HR)), dyspnea (Borg scale) and peripheral oxygen saturation (SpO_2_) were determined. During the tests, HR and SpO_2_ were monitored continuously with the aid of an oximeter (Model 9500, Nonin Medical Onyx^®^). The UULEX and 6MWT were performed on three consecutive days, with a maximal evaluation time of 90 min during each day.

#### Respiratory muscle strength and endurance assessment

Respiratory muscle strength was assessed using a compound pressure gauge (MV-150/300, Record^®^—Ger-Ar Comércio de equipamentos Ltda) for the acquisition of maximal inspiratory and expiratory pressure (PImax and PEmax, respectively), and was carried out according to the guidelines for the pulmonary function test described by Pereira and Neder [10]. At least three consecutive satisfactory measurements were conducted, with the largest value used in analysis, provided there was less than a 5 % difference between the two highest values. After the measurements, the PImax and PEmax values were expressed as a percentage of the predicted value, using the equations proposed by Pereira and Neder [10]. Respiratory muscle endurance was assessed using a modified version of the Johnson et al. protocol (incremental threshold loading) [18]. For these measurements, linear pressure load resistors were used for the inspiratory muscles (Threshold IMT^®^–Philips Respironics) and expiratory muscles (Threshold PEP^®^–Philips Respironics). The predetermined initial loads for the inspiratory and expiratory muscles were 10 cmH_2_O and 5 cmH_2_O, respectively. A load of 5 cmH_2_O was added every 2 min. The final loads sustained for at least 1 min were considered the maximal inspiratory and expiratory loads (ILmax and ELmax, respectively), with the total duration of the test representing the endurance time. The inspiratory and expiratory limit times (IT-lim and ET-lim, respectively) were determined by the maximal time over which the participant was able to sustain 80 % of ILmax or ELmax. HR, RR, and SpO_2_ were monitored during the test.

#### Quality of life and dyspnea assessment

Quality of life was assessed through administration of the saint george’s respiratory questionnaire (SGRQ), which is a standardized questionnaire specific to patients with COPD and asthma, that has been validated for the Brazilian population [19]. The SGRQ has three domains: symptoms, activity, and impact. The total score (SGRQ_Total_) was also calculated, ranging from 0 (best health status) to 100 (worst health status). These domains appear to be valid for both genders, a broad age range, and all levels of COPD severity [19]. Dyspnea was assessed using the Portuguese-language version of the Medical Research Council (MRC) scale [20]. In this assessment, patients report their subjective degree of dyspnea on a scale of 1–5, based on the following classifications: shortness of breath during intense exercise (1); shortness of breath when walking quickly or walking up a slightly inclined ramp (2); walking slower than others the same age due to shortness of breath or stopping to breathe even when walking slowly (3); resting to breathe after walking less than 100 m or after a few minutes (4); and no longer leaving home due to shortness of breath, or shortness of breath when getting dressed (5) [20].

### Statistical analysis

Statistical analysis was performed with R-2.8.0 and GraphPad Prism 5.01 (GraphPad-Scientific Softwares). A sample size of 54 subjects was determined to be necessary for a minimum detectable effect of 0.18, based on a significance of 5 % and a power of 70 %. The Kolmogorov–Smirnov test was used to determine the normality of the distribution for all variables. The paired Student’s *t* test and Mann–Whitney test were used for comparisons between groups for variables with normal and non-normal distributions, respectively. For multivariate analyses using multiple linear regressions, covariates with a significant association (5 %) with the dependent variable were considered. The Kolmogorov–Smirnov test revealed that the 6MWD had a non-normal distribution. Therefore, a Box-Cox transformation was performed with a cubic mean equation proposed for this variable, using the following terms: Y_2_ = UULEX, Y_2_ = SGRQ total and Y_3_ = 6MWD. (dependent variables), and a vector X = (x_1_, x_2_, …, x_18_) (independent variables). The following mathematic equation was proposed: $\mu = \beta_{0} + \beta_{ 1} x_{ 1} + \beta_{ 2} x_{ 2} + \cdots + \beta_{n} x_{n}$.

In this equation, *µ* is the mean of the chosen dependent variable, $\beta_{0}$ is the intercept, $\beta_{ 1} {\text{ to }}\beta_{n}$ are the effects of each dependent variable in relation to the Y variable, and $x_{i}$ is the value of the independent variable, *i*, selected to be tested in the model. The residuals of all final models were determined in relation to a normal distribution. Moreover, all other assumptions of the regression model were satisfied.

## Results

Fifty-four patients were included in the study, among these, 44 (81.5 %) had moderate to severe COPD (23 [52.3 %] of the men and 21 [47.7 %] of the women) and 10 (18.5 %) had very severe COPD (7 [7 %] of the men and 3 [3 %] of the women).

Based on the results from the MINI diagnostic tool, 12 patients (22.2 %) from 54 COPD patients had depression (depression_(MINI)_). In addition, among COPD patients diagnosed with depression by MINI, 10 (83.3 %) had moderate to severe COPD and only 2 (16.7 %) had very severe COPD. No significant differences were found between depressive and non-depressive patients with regard to anthropometric and lung function variables (Table 1). For the demographic and socioeconomic variables (Table 2), patients with depression_(MINI)_ reported insomnia (75 %, n = 9), and hospitalization in the previous year (58.3 %, n = 7), occurring primarily due to exacerbation of COPD [1] and other causes.

**Table 1:** Descriptive and comparative analysis of anthropometric, lung function variables and strength and endurance of the respiratory muscles

Variable	Total	Men	Women	p value	Non-depressive	Depressive	p value
	n = 54	n = 30	n = 24		n = 42	n = 12	
Age (years)	68.93 ± 9.03	71.40 ± 8.10*	65.83 ± 9.34	0.0109	70.10 ± 1.25	64.83 ± 3.21	0.075
BMI (kg/m^2^)	25.31 ± 5.53	23.71 ± 3.62	27.31 ± 6.81*	0.0326	24.76 ± 0.82	27.23 ± 1.74	0.176
FVC (% of predicted)	66.92 ± 14.84	65.83 ± 15.46	68.29 ± 14.24	0.8918	65.97 ± 2.09	70.25 ± 5.47	0.384
FEV_1_ (% of predicted)	45.41 ± 15.90	41.20 ± 15.76	50.67 ± 14.76	0.1346	43.55 ± 2.28	51.92 ± 5.31	0.108
FEV_1_/FVC (%)	50.57 ± 11.77	45.90 ± 11.56	56.42 ± 9.30*	0.0121	0.64 ± 0.02	0.73 ± 0.042	0.105
PImax (% of predicted)	85.76 ± 24.02	83.60 ± 22.01	88.45 ± 26.55	0.7695	85.24 ± 3.28	87.58 ± 9.52	0.768
PEmax (% of predicted)	117.7 ± 30.19	113.9 ± 21.73	122.4 ± 38.26	0.3854	85.24 ± 3.28	87.58 ± 9.52	0.768
ILmax (cmH_2_O)	19.91 ± 7.42	21.00 ± 7.58	18.54 ± 7.14	0.2182	20.71 ± 1.13	17.08 ± 2.08	0.116
IT-lim (seconds)	341.5 ± 190.8	351.9 ± 184.6	328.6 ± 201.5	0.5539	365.8 ± 30.03	256.7 ± 44.85	0.107
ELmax (cmH_2_O)	19.17 ± 9.20	21.67 ± 8.84*	16.04 ± 8.84	0.0312	20.48 ± 1.41	14.58 ± 2.34	0.057
ET-lim (seconds)	366.1 ± 143.3	376.8 ± 137.4	352.8 ± 152.3	0.7240	377.2 ± 20.96	327.1 ± 48.40	0.289

Data expressed as mean ± standard deviation

*BMI* body mass index, *FVC* forced vital capacity (post-bronchodilator), *FEV*_*1*_ forced expiratory volume in the first second post-bronchodilator, *PImax* maximal inspiratory pressure, *PEmax* maximal expiratory pressure, *ILmax* maximal inspiratory load, *ELmax* maximal expiratory load, *IT-lim* inspiratory limit time, *ET-lim* expiratory limit time

**Table 2:** Description of patients according to demographic/socioeconomic variables

Variable	Total	Men	Women	Non-depressive	Depressive
	n = 54 (%)	n = 30 (%)	n = 24 (%)	n = 42 (%)	n = 12 (%)
Monthly income					
Less than the minimum salary	33.3	23.3	45.8	28.6	50.0
1–5 times the minimum salary	57.4	60.0	54.2	59.5	50.0
5–10 times the minimum salary	9.3	16.7	0	11.9	0
Greater than 10 times the minimum salary	0	0	0	0	0
Schooling					
0–2 years	24.2	16.7	33.4	23.8	25.0
3–4 years	40.7	40.0	41.6	38.1	50.0
5–8 years	12.9	20.0	4.2	14.3	8.3
9 years or more	22.2	23.3	20.8	23.8	16.7
Smoking habits					
Never smoked	0	0	33.4	0	0
Ex-smoker	79.6	86.7	41.6	83.3	66.7
Smoker	7.4	10.0	4.2	14.3	8.3
Ex-passive smoker	12.9	3.3	20.8	23.8	25.0
Family support					
Lives alone	9.2	3.3	16.7	7.1	16.7
Lives with family in positive manner	85.2	90.0	79.1	90.4	66.6
Lives with family in negative manner	5.6	6.7	4.2	7.1	16.7
Pulmonary rehabilitation					
Yes	46.3	40.0	54.2	47.6	58.3
No	53.7	60.0	45.8	52.4	41.7
Hospitalization in the previous year					
Yes	48.2	53.3	41.6	45.3	58.3
No	51.8	46.7	58.4	54.7	41.7
Insomnia					
Yes	48.2	43.4	54.2	40.4	75.0
No	51.8	56.6	45.8	59.6	25.0
Dyspnea grade					
1	3.8	6.6	0	4.7	0
2	12.9	13.3	12.5	16.6	0
3	20.4	20.0	20.8	19.1	25.1
4	24.1	20.1	29.2	21.4	33.3
5	38.8	40.0	37.5	38.2	41.6

Regarding dyspnea, Grade 3 was reported in 25.1 %, Grade 4 in 33.3 %, and Grade 5 in 41.6 % of the patients with depression_(MINI)_.

**Fig. 2:**
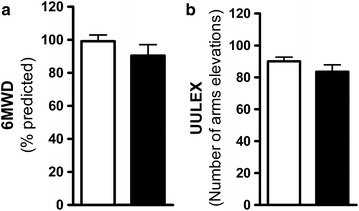
Influence of depression diagnosed by the MINI on functional capacity. Functional capacity as estimated by (**a**) a six-minute walk test (6MWT), and by (**b**) an unsupported upper-limb exercise test (UULEX). The *light bars* show the data of the non-depressive patients and the *black bars* show the data of the patients diagnosed with depression

**Fig. 3:**
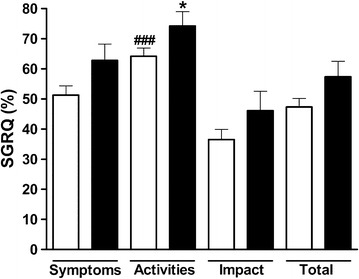
Influence of depression diagnosed by the MINI on SGRQ domains. The *light bars* show the data of the non-depressive patients and the *black bars* show the data of the patients diagnosed with depression. *Asterisk*, *bash* Indicates statistical significance compared to SGRQ activity with impact domain in Depressive patients and non-depressive patients, respectively, (*p < 0.05 and ^###^p < 0.001, one-way ANOVA followed by the Bonferroni’s multiple comparison test)

Functional capacity showed no significant difference between depressed and non-depressed patients, as assessed by 6MWD (90.43 ± 6.6 and 99.15 ± 3.8 meters, respectively p = 0.279) (Fig. 2a) and UULEX (83.58 ± 4.2 and 90.07 ± 2.6 number of arms elevations, respectively p = 0.239) (Fig. 2b). However, the score in the SGRQ activity domain was significantly higher than the impact domain for patients with depression_(MINI)_, when compared to those without depression_(MINI)_ (74.23 ± 16.38 and 46.09 ± 22.35 %, respectively p > 0.05) (Fig. 3). The best models for the dependent variables UULEX, SGRQ_Total_, and 6MWT are summarized in Table 3. The covariates SGRQ_Total_ and gender (p = 0.024 and p = 0.028, respectively; R^2^ = 16.7 %) were significant in explaining the response variable UULEX. Age, monthly income, insomnia, and the 6MWD were significant in explaining the SGRQ_Total_ score (p = 0.001, p = 0.006, p = 0.000, and p = 0.000, respectively, R^2^ = 46 %). FEV1 (percentage predicted post-bronchodilator) and gender were significant in explaining the 6MWD results (p = 0.003 and p = 0.000 respectively, R^2^ = 22 %). Interestingly, depression_(MINI)_ did not significantly explain any of the dependent variables (UULEX versus depression_(MINI),_ p = 0.592; SGRQ_Total_ versus depression_(MINI),_ p = 0.094; 6MWT versus depression_(MINI),_ p = 0.271).

**Table 3:** Result of best models for the dependent variables UULEX, SGRQ, and 6MWD

Variable	Covariates	Estimate	Standard error	t statistic	p value	VIF	Adjusted R^2^
UULEX (# of arm elevations)	Intercept	288.95	29.68	9.73	0.000**		0.167
	Gender (male)	39.63	17.50	2.26	0.028*	1.02	
	SGRQ_TOTAL_	−1.17	0.50	−2.33	0.024*	1.02	
SGRQ_TOTAL_	Intercept	129.57	17.49	7.40	0.000**		0.460
	Age (years)	−0.69	0.20	−3.35	0.001**	1.13	
	Monthly income (>1 minimum salary)	−11.35	3.96	−2.86	0.006**	1.18	
	Insomnia (yes)	13.37	3.70	3.61	0.000**	1.09	
	6MWD (meters)	−0.07	0.02	−3.53	0.000**	1.41	
6MWD (meters)	Intercept	1.76E + 07	2.15E + 07	0.818	0.417		0.22
	Gender (male)	4.37E + 07	1.22E + 07	3.577	0.000**	1.14	
	FEV_1_(% pred post-BD)	1.21E + 06	3.92E + 05	3.076	0.003**	1.14	

Values correspond to beta coefficients calculated at two levels of significance: * p < 0.05 and ** p < 0.01; VIF refers to multicollinearity between independent variables, for which values greater than one indicate this condition

*UULEX* unsupported upper-limb exercise test, *SGRQ*_*TOTAL*_ total score on Saint george’s respiratory questionnaire, *6MWD* six-minute walking distance, *FEV1 (% pred post-BD)* % of predicted forced expiratory volume in the first second following the use of a bronchodilator

Depression prior to evaluations, as reported from patient self-perception, was observed in 16.7 % of the men and 50 % of the women. However, diagnosis using the MINI for depression was positive in 6.6 % of the men and 41.6 % of the women. This shows that depression_(MINI)_ was more prevalent among women.

The men were older, taller, and had higher ELmax (cmH_2_O) values than women, while women had higher BMI and FVC/FEV_1_ (%) values (Table 1). Insomnia was reported in 43.3 % of the men and 54.1 % of the women (Table 2).

The percentage of men and women in relation to the different degrees of dyspnea is summarized in Table 2. No patient had any exacerbations of COPD in the 6 months prior to the tests. However, 53.3 % of the men and 41.6 % of the women reported hospitalizations in the previous year. Thirty percent (30 %) of the men and 54.2 % of the women reported a family history of depression.

Regarding the SGRQ domains and the functional capacity variables (6MWD and UULEX), a significant gender difference was observed in the 6MWD (meters) (p = 0.005) and UULEX (p = 0.016), but not for the SGRQ (Table 4).

**Table 4:** Descriptive and comparative analysis between genders for quality of life and functional capacity variables

Variable	Total	Males	Females	(p value)
	n = 54	n = 30	n = 24	
SGRQ_SYMPTOMS_ (%)	53.85 ± 20.22	53.41 ± 19.70	54.39 ± 21.26	0.766
SGRQ_ACTIVITY_ (%)	66.44 ± 17.65	63.78 ± 17.62	69.75 ± 17.49	0.238
SGRQ_IMPACT_ (%)	38.63 ± 22.15	34.51 ± 21.45	43.78 ± 22.38	0.259
SGRQ_TOTAL_ (%)	49.59 ± 18.42	46.53 ± 17.95	53.41 ± 18.66	0.244
6MWD (meters)	445.2 ± 95.81	468.2 ± 105.0*	416.3 ± 75.44	0.005
6MWD (% of predicted)	97.21 ± 24.40	101.6 ± 27.24	91.74 ± 19.48	0.123
UULEX (# of arm elevations)	257.2 ± 82.15	281.9 ± 89.26*	226.4 ± 61.02	0.016

Data expressed as mean ± standard deviation

*SGRQ* saint george’s respiratory questionnaire—total score and scores on symptoms, activity, and impact domains, *6MWD* six-minute walking distance, *UULEX* unsupported upper-limb exercise test

Therefore, our results show that the MINI diagnosed depression in 22.2 % of patients analyzed. Among depressive patients, most were female. Despite the fact that the MINI was effective for diagnosing depression, no influence from depression, as diagnosed by the MINI, was observed on the quality of life or the functional capacity of the COPD patients analyzed.

## Discussion

Previously, studies observing patients with COPD have shown that patients with depression and anxiety exhibit poorer quality of life and functional capacity [21, 22]. In this study, using the MINI as a diagnostic tool for depression, no association was found between depression_(MINI)_ and functional capacity, as assessed by submaximal exercise tests, 6MWT and UULEX, or quality of life, as assessed by SGRQ_Total_, in patients with moderate to very severe COPD. Thus, our results suggest that the method used for the identification of depression may be important for ascertaining the impact of depression on functional capacity and quality of life among patients with COPD.

The majority of previous studies have shown that, among patients with COPD, depression is diagnosed twice as often as identified in this study [23], and that COPD consistently increases the risk of depression [24]. Several factors may be associated with depression in patients with COPD [25], including increased dyspnea, a reduction in exercise capacity [4], less social support [26], lower levels of education [27], and a lower BMI [4]. We believe that differences between our results and those from previous studies are due to the methodology used in identifying the presence of depression in COPD patients; specifically, using tools that focus on the symptoms, rather than the diagnosis. Under these circumstances, we suggest that many studies overestimate the presence of depression as a comorbidity of COPD.

Additionally, the diagnosis of depression in patients with COPD is complicated by the overlap of symptoms from both diseases [28], and the suggestion that depression may result from COPD is highly controversial [22, 24]. This shows the importance of studies that propose to validate tools enabling efficient identification of the presence or absence of depression as in COPD patients.

In the present study, we used the MINI as a diagnostic tool, as it is a structured diagnostic interview for depression, and is compatible with the diagnostic criteria of the DSM-IV and ICD-10. In addition, it has been validated in the Portuguese language, and is considered the standard for confirmation of the presence or absence of anxiety disorders in COPD patients. To our knowledge, no published studies exist that have used the MINI as an assessment methodology in patients with COPD [11, 29].

The results obtained show that depression_(MINI)_ was identified in only 22.2 % of patients included in this study, in contrast to other studies that show a large incidence of depression among patients with COPD [23]. Although the percentage of patients with depression in our sample was relatively small (22.2 %), depression_(MINI)_ was significantly more prevalent among women (n = 10, 41.7 %) than in men (n = 2, 6.6 %). Interestingly, our results are consistent with those obtained in a recent study evaluating the influence of gender on risk factors for COPD. It was noted that women with COPD have more concomitant depression than men. However, the influence of gender on COPD is complex and involves several other factors, including the sensitivity difference of tobacco effects, anatomical, hormonal and behavioral changes, and differential response to therapy [30]. In addition, another factor that may have contributed to a greater number of women with depression_(MINI)_ in our sample is the small sample size and selection of patients based on convenience.

The negative influence of depression on functional capacity and quality of life appears to be greater than influences determined by the severity of COPD [7]. However, the association between depression and COPD severity remains unclear [31]. A recent systematic review of prospective cohort studies concluded that anxiety and depression predict health-related quality of life in COPD patients [22]. However, this longitudinal analysis did not show a causal relationship between depression and anxiety and future health-related quality of life [22]. According to other studies, depressive patients typically exhibit a poorer quality of life, greater social isolation, and less adherence to treatment [32]. Depressive patients also have a 2.8-fold greater likelihood of experiencing COPD exacerbation, longer hospital stays [33], and possibly a higher mortality rate [26], than non-depressive patients. However, findings from a recent study suggest that the relationship between patient characteristics, common symptoms of depression, and quality of life differ when disease-specific and generic measures of quality of life are evaluated [34].

In this study, we used the SGRQ; a validated, disease-specific questionnaire widely used in evaluating the quality of life for COPD patients in clinical trials. This questionnaire consists of 50 items measuring 3 domains: symptoms (8 items), activity (16 items), and impact (26 items) [19]. Other authors have observed that SGRQ_(Activity)_, SGRQ_(Impact)_, and SGRQ_(Total)_ scores were significantly worse in COPD patients who were depressive [35, 36]. However, in the present study, only the SGRQ_(Activity)_ domain score was significantly worse in patients with depression_(MINI)_. Further, analysis of dependent variables showed that depression did not correlate with the SGRQ_(total)_.

Psychometric differences between the SGRQ domains are recognized in the literature, and it has been suggested that the SGRQ_(Activity)_ and SGRQ_(Symptoms)_ domains have greater accuracy [37]. In present study, depression was identified using the MINI diagnostic tool, where the influence from the patient’s subjective evaluation of their health is mitigated. For this reason, we believe that there was a difference in only the SGRQ_(Activity)_ domain, because this domain is more accurate and less vulnerable to influences from patient subjectivity.

Exercise tests are often performed to assess functional impairment and disease severity, as well to establish a prognosis and determine the etiology of intolerance to exercise [1]. A recent multiple regression analysis revealed that lower SGRQ_(Total)_ scores, higher depression scores, a longer time needed to complete the Timed Up and Go test at baseline, and increases in dyspnea during a 1-year follow-up period, were predictors of deterioration in the disease-specific health status of patients with advanced COPD [38].

In the present study, we used two exercise tests, the UULEX and the 6MWT, to measure the functional capacity of COPD patients and the influence of depression, as diagnosed by the MINI, on the performance of patients.

Regarding the upper limbs, two possible mechanisms may be related to these findings. Firstly, there may be neuromechanical dysfunction (thoracoabdominal asynchrony) of the respiratory muscles (diaphragm and accessory muscles). Secondly, there may be changes in lung volume during activities involving the upper limbs [39]. According to Lebzelter et al. [39], limitations of patients with COPD during upper limb exercises without support are predominantly dependent upon respiratory muscle function. Further, inspiratory capacity, functional residual capacity, FEV_1_, and arm circumference explained 77 % of the variation in exercise time during the UULEX test.

In the present study, however, only the combination of the covariates SGRQ_(Total)_ and gender were significant in explaining the response variable UULEX, although correlation was weak (R^2^ = 16.7 %). Further, no significant differences in upper limb endurance or respiratory muscle endurance between patients with and without depression_(MINI)_. To our knowledge, there are no studies on the association between depression and upper limb or respiratory muscle endurance. Additionally, in a recent study, no significant relationships were observed between psychological factors and daily activities [40].

The main variables related to low exercise capacity, measured by the 6MWT, are isometric strength of the quadriceps [41], PImax [41], FEV_1_ [42], FVC [43], dyspnea [44], body weight [41], blood pressure, arterial carbon dioxide pressure [43], depression [45], age [45], and gender [45]. In the present study, the explanatory power of the regression equation for functional capacity was weak (R^2^ = 22 %) and only FEV_1_ and gender were significant in explaining the trends seen in our 6MWT results.

In accordance with trends reported in studies by Borak et al. and Light et al. [43, 46], the present study found no association between depression and the 6MWD. In contrast, other studies show an association between a worse performance in the 6MWD and depression [47] and a reduced daily and maximal exercise capacity, compared to non-depressive patients. Further, after adjustment for age and severity of symptoms, it was found that poor health status, severity of COPD, and depression were the main factors associated with a low walking times (<30 min/day) in COPD patients [48].

Symptoms, functional limitations, and social and psychological impact are among the factors affecting the quality of life of patients with COPD [40]. In the present study, age, monthly income, insomnia, and the 6MWD were significant in explaining the quality of life among the COPD patients. However, the diagnosis of depression by the MINI was not a significant factor in explaining possible changes in the quality of life, as measured by SGRQ, and functional capacity, as measured by UULEX and the 6MWT tests, in COPD patients evaluated in this study. The fact that we used a diagnostic tool for identifying depression, as opposed to diagnosis based on symptoms, may explain the lack of correlation between depression, quality of life, and functional capacity in COPD patients. We suggest that this is primarily because the tools used when identifying depression via symptoms may be vulnerable to confounding factors.

Some details of the present study that may help in understanding the results are described as follows. This study used a structured interview for the diagnosis of depression, performed by only one trained psychiatrist, who was blinded to the evaluation results from the functional tests performed by the physiotherapist or the pulmonary function results performed by the pulmonologist. In addition to the structured interview, this study also administered a quality of life questionnaire specific to COPD. Moreover, a broad-scoped assessment of functional capacity was performed by a physical therapist who had no knowledge regarding the results of the psychiatric evaluation. This study used a population from a clinical setting in a cross-sectional study. Some limitations exist in this study. Because this was a cross-sectional study, it was not possible to establish causal relationships to support the existence of a temporal sequence between the exposure factor and the subsequent development of the disease. Additionally, this work was carried out with a sample of convenience, which may limit the ability of our results to be generalized.

## Conclusion

Despite a high proportion of depression in COPD patients, especially in women, depression, as diagnosed by the MINI, was not correlated with functional capacity test results or quality of life in patients with moderate to very severe COPD. This suggests that depression_(MINI)_ appears to be appropriate to identify depression in the population studied, thus contributing to a better management of patients with COPD.

## Abbreviations

COPD: Chronic Obstructive Pulmonary Disease
FEV_1_: Forced expiratory volume in one second
FVC: Forced vital capacity
MINI: Mini international neuropsychiatric interview
BMI: Body mass index
ATS: American thoracic society
UULEX: Unsupported upper-limb exercise
6MWT: Six-minute walk test
6MWD: Six-minute walk distance
RR: Respiratory rate
HR: Heart rate
PImax: Maximal inspiratory pressure
PEmax: Maximal expiratory pressure
ITL: Incremental threshold loading
ILmax: Maximal inspiratory load
ELmax: Maximal expiratory load
IT-lim: Inspiratory limit time
ET-lim: Expiratory limit time
SGRQ: Saint George’s Respiratory Questionnaire
MRC: Medical Research Council
